# Integrin αvβ5 is a primary receptor for adenovirus in CAR-negative cells

**DOI:** 10.1186/1743-422X-7-148

**Published:** 2010-07-08

**Authors:** Cynthia Lyle, Frank McCormick

**Affiliations:** 1 UCSF Helen Diller Family Comprehensive Cancer Center, University of California, San Francisco, San Francisco, CA, 94158, USA

## Abstract

**Background:**

Viruses bind to specific cellular receptors in order to infect their hosts. The specific receptors a virus uses are important factors in determining host range, cellular tropism, and pathogenesis. For adenovirus, the existing model of entry requires two receptor interactions. First, the viral fiber protein binds Coxsackie and Adenovirus Receptor (CAR), its primary cellular receptor, which docks the virus to the cell surface. Next, viral penton base engages cellular integrins, coreceptors thought to be required exclusively for internalization and not contributing to binding. However, a number of studies reporting data which conflicts with this simple model have been published. These observations have led us to question the proposed two-step model for adenovirus infection.

**Results:**

In this study we report that cells which express little to no CAR can be efficiently transduced by adenovirus. Using competition experiments between whole virus and soluble viral fiber protein or integrin blocking peptides, we show virus binding is not dependent on fiber binding to cells but rather on penton base binding cellular integrins. Further, we find that binding to low CAR expressing cells is inhibited specifically by a blocking antibody to integrin αvβ5, demonstrating that in these cells integrin αvβ5 and not CAR is required for adenovirus attachment. The binding mediated by integrin αvβ5 is extremely high affinity, in the picomolar range.

**Conclusions:**

Our data further challenges the model of adenovirus infection in which binding to primary receptor CAR is required in order for subsequent interactions between adenovirus and integrins to initiate viral entry. In low CAR cells, binding occurs through integrin αvβ5, a receptor previously thought to be used exclusively in internalization. We show for the first time that integrin αvβ5 can be used as an alternate binding receptor.

## Background

Viruses bind to specific cellular receptors to infect their hosts. The specific receptors a virus uses are important factors in determining host range, cellular tropism, and pathogenesis. HIV-1 is one of the best characterized viruses in terms of viral entry. HIV-1 first binds to CD4, its primary receptor [1,2]. Although CD4 binding was initially thought to be sufficient for infection, it was later found that a second interaction between HIV and chemokine co-receptors CCR5 or CXCR4, is also required [3-5]. Binding to CD4 occurs first, triggering conformational changes in the HIV protein gp120, revealing the previously hidden binding site for its co-receptors, which then trigger membrane fusion [6,7]. The discovery of HIV's requirement for co-receptors in addition to CD4 represented a significant shift in our understanding of viral entry. The idea that a single virus bound to a single entry receptor was replaced with the idea that viral entry is the result of distinct sequential events requiring multiple surface proteins.

In keeping with this multistep entry model, adenoviruses have been proposed to use a primary receptor to mediate binding and co-receptors to mediate internalization [8]. Adenoviruses are non-enveloped double stranded DNA viruses associated with respiratory disease, ocular disease, and gastroenteritis [9]. Adenoviruses have three major capsid proteins: hexon, which forms the bulk of the capsid and is present in 240 copies, penton base, which is present in five copies at each of the twelve vertices, and fiber, a homotrimeric protein that protrudes from each vertice, extending outward from the penton base. More than 50 human serotypes of adenovirus have been identified to date [10,11]. The best studied of these are the species C adenoviruses, including Adenovirus Serotype 2 (Ad2) and Adenovirus Serotype 5 (Ad5). The primary receptor for species C adenoviruses is thought to be Coxsackie and Adenovirus Receptor (CAR), which binds to the globular knob domain of fiber [12]. This high affinity interaction docks the virus to the cell, thus allowing secondary interactions to occur. Following fiber binding to CAR, the penton base engages αvβ3 and αvβ5 integrins to initiate endocytosis and viral entry [8]. Adenoviruses bind to integrins via an RGD motif present in the penton base. The penton base-integrin interaction is proposed to be exclusively involved in virus internalization and not to contribute to virus binding [8].

Several studies have reported alternate mechanisms for adenovirus entry. Huang et al demonstrated that adenovirus binds to hematopoietic cells via a penton base interaction with Integrin αMβ2, an integrin not expressed on epithelial cells, but still requires αv integrins for virus internalization [13]. Additionally, Ad5 has also been proposed to use heparan sulphate glycosaminoglycans as receptors [14,15] and to use lactoferrin as a bridge between viral particles and the cell surface [16,17]. In both of these systems, adenovirus fiber is the viral protein required for binding. Further complication is observed in vivo. Infection of liver cells, which has been well characterized, is CAR-independent and instead depends on adenovirus hexon binding the blood coagulation factor F(X) which leads to infection [18-24]. Additionally, in both mice and non-human primates, adenoviruses with mutant fibers ablated for CAR binding show a similar biodistribution compared to wild type viruses [18,21,25,26]. Similarly, a lack of correlation between CAR expression and adenovirus infection has been observed in cell lines, though the mechanism by which infection of cells with low CAR is achieved is undefined [12,27,28].

We also observe a lack of correlation between CAR expression and infection in cell lines. Indeed, we report here that an Ad5 vector can efficiently transduce cancer cells which express little to no CAR. Further, Ad5 binds to these cells via integrin αvβ5, a surface protein previously thought to be used exclusively for internalization and thus classified as a secondary co-receptor. These observations lead us to further question the two-step model for adenovirus infection, in which adenovirus must first bind to CAR, the primary receptor, in order to bind to integrins and trigger viral entry.

## Results

### Adenovirus infection is variable across a panel of cancer cell lines

**Figure 1:**
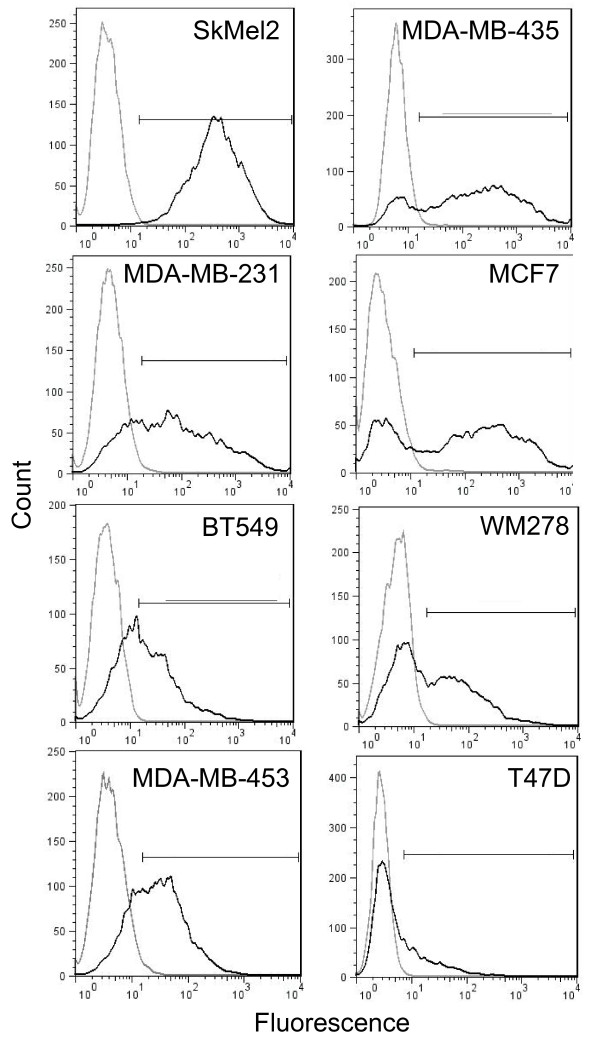
**Ad5 transduction is variable across a panel of cancer cell lines**. Cells were infected with Ad5-GFP at MOI (PFU/cell) 25 and incubated overnight. Infection was quantified immediately following the overnight incubation using flow cytometry analysis of cells infected with Ad5-GFP (black line) compared to an uninfected control (grey line) for each cell line. 10000 events were acquired and live cells were gated before assessing fluorescence. The percentage of cells positive for GFP is quantified as well as the mean fluorescence intensity (MFI) in Table 1. Data shown is representative of at least two independent experiments.

Although alternate entry routes have been described, the best characterized model of adenovirus entry requires binding of adenovirus fiber to the cellular membrane protein CAR to initiate infection [12]. CAR is a cell adhesion molecule and, like other cell adhesion molecules, is down-regulated during cancer progression [29-34]. Several reports have shown that the ability of adenoviruses to infect different cancer cell lines is variable [31,33-36]. Therefore, we chose a panel of cancer cell lines, consisting of human melanoma cells and human breast cancer cells, to study the requirement for a CAR-mediated binding event in adenovirus infection. We first measured the ability of Ad5 to infect this panel of cancer cell lines. Cells were infected with a non-replicating virus deleted for E1A that expresses GFP (Ad5-GFP) and GFP expression was used as a measure of transduction. Cells were infected at a multiplicity of infection (MOI) 25 because at this MOI cells show a high level of transduction but the system is not saturated (Additional File 1). Figure 1 shows that at MOI 25, Ad5-GFP transduced these cells with a wide range of efficiency. The percentage of cells positive for GFP as well as the mean fluorescence intensity (MFI) for each cell line is displayed in Table 1. SkMel2 cells were most infectible with 97% of cells positive for GFP. MDA-MB-435, MCF7, MDA-MB-231, and MDA-MB-453 cells were infected at an intermediate level, ranging from 58-79% of cells GFP-positive. On the lower end of the spectrum, Ad5-GFP infected only about 40% of both BT549 and WM278 cells. Finally, T47D cells show a very small shift in fluorescence after infection, indicating these cells are resistant to Ad5 infection.

**Table 1:** Quantification of Ad5 transduction and CAR expression across the panel of cell lines

	Transduction		CAR Expression	
**Cell Line**	**% positive**	**MFI**	**% positive**	**MFI**

SkMel2	97.1	575	98.3	68.3

MDA-MB-231	69.3	438	93.5	31.2

BT549	35.5	86.2	42.2	12.6

MDA-MB-453	58.1	78.3	98.1	40

MDA-MB-435	79.2	657	0.5	3.9

MCF7	67	673	2.9	4.9

WM278	45.9	186	2.8	12.6

T47D	13.6	50.6	71.8	32.2

**Figure 2:**
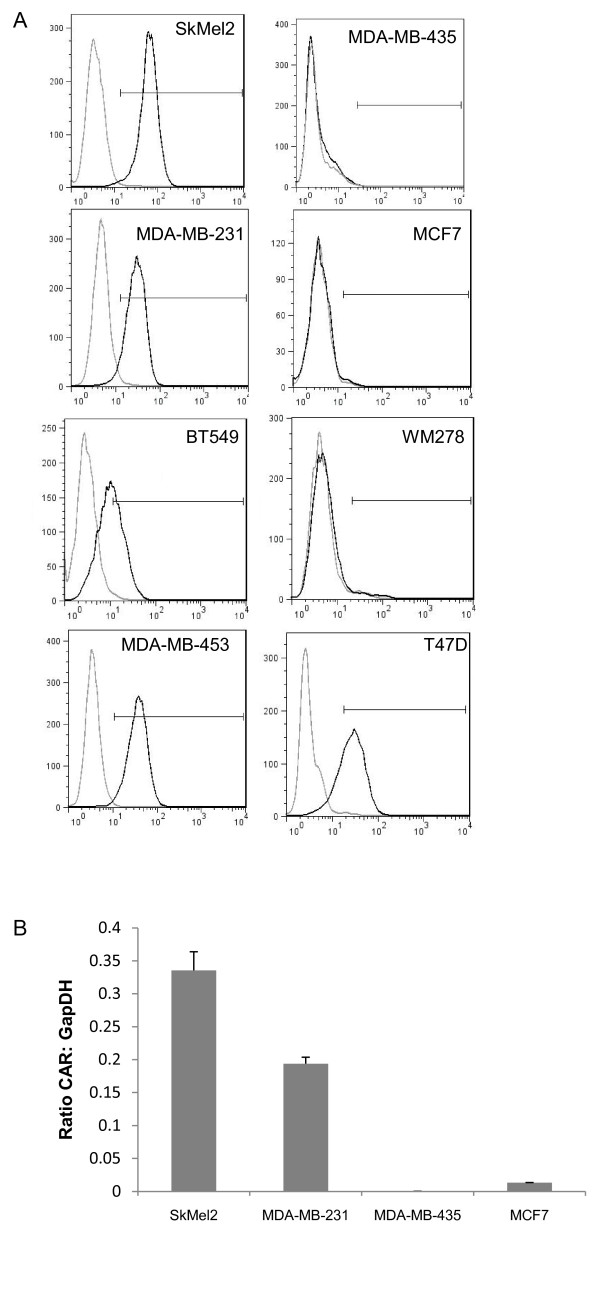
**CAR expression levels**. (A) Surface CAR levels were detected using flow cytometry. Cells were stained either with the monoclonal antibody RmcB which recognizes CAR (black line) or for control, only the secondary antibody, goat anti-mouse Alexa 488(grey line). The secondary only population was set to approximately 1% positive and used as the negative population for each cell line. The percentage of cells which are positive for CAR as well as the MFI is quantified and displayed in Table 1. Data shown is representative of at least two independent experiments. (B) CAR mRNA levels were determined using quantitative real-time PCR. CAR mRNA levels are compared to the control gene GapDH and data is presented as a ratio of CAR: GapDH. Data is the average of at least two independent experiments and error bars represent standard deviation.

Quantification of data displayed in Figure 1 (Transduction) and Figure 2a (CAR expression). MFI is mean fluorescence intensity.

### Surface CAR levels do not explain differences in Ad5 entry

CAR expression is a primary determinant for Ad5 entry. To investigate whether the variability in transduction could be explained by differences in CAR levels, we next measured surface CAR expression using flow cytometry. Figure 2a shows that most of the cell lines, including SkMel2, MDA-MB-231, MDA-MB-453 and T47D cells, express CAR on the majority of cells. Interestingly, T47D cells, which are resistant to Ad5 infection, also express CAR on the cell surface. In contrast, WM278, MDA-MB-435, and MCF7 cells, all of which are infectible with Ad5-GFP, express little to no CAR on the cell surface. Quantification of both the percentage of cells positive for CAR expression as well as the MFI is displayed in Table 1. These data suggests CAR binding is neither sufficient nor necessary for Ad5 entry.

We next verified CAR expression by measuring mRNA levels in two high CAR and two low CAR cell lines using TaqMan analysis. Figure 2b shows that CAR mRNA levels correlated with surface protein levels (Figure 2b). The two cell lines (MDA-MB-435, and MCF7) which show little to no surface CAR expression also had very little to, in the case of MDA-MB-435 cells, no detectable CAR mRNA expressed (Figure 2b).

### Infection of CAR-negative cells is fiber-independent

**Figure 3:**
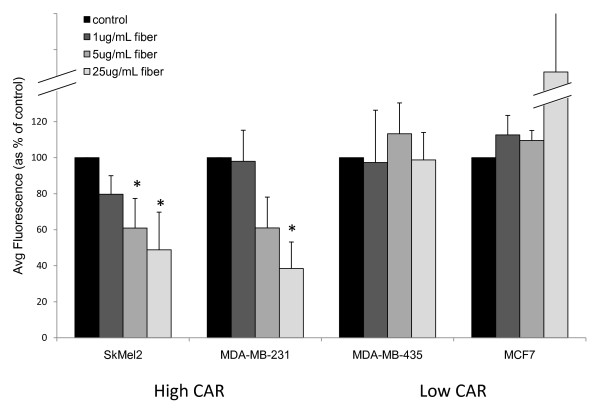
**Infection low CAR cells is fiber-independent**. Cells were preincubated for 1 hr with increasing concentrations of soluble fiber followed by addition of Ad5-GFP and further incubation overnight. Flow cytometry was used to quantify fluorescence intensity. Data shown is the average of at least three independent experiments and error bars represent standard deviation. * indicates statistical significance, p ≤ 0.05.

Previous reports have indicated that the fiber-CAR interaction mediates binding of Ad5 to the cell surface [8,12,37]. Additionally, Ad5 fiber has been reported to bind cells through heparin sulphate proteoglycans or through a lactoferrin bridge [14-17]. Therefore, we next examined whether Ad5 infection, although not dependent on CAR, is still dependent on fiber, perhaps by binding to a different cellular receptor. To address this question, we tested the dependence of infection in low CAR cells on fiber. Cells were preincubated with soluble fiber prior to adding Ad5-GFP to the cells and measuring transduction, as determined by GFP expression. Transduction of MDA-MB-231 and SkMel2 cells, both of which express CAR (Figure 2), could be blocked in a dose-dependent manner by preincubation with soluble fiber (Figure 3). In contrast, transduction of low CAR cells MCF7 and MDA-MB-435 was not blocked. Ad5 infection of MCF7 and MDA-MB-435 cells is therefore not only CAR-independent but also fiber-independent. Interestingly, at the highest concentration, preincubation with fiber actually increases transduction in MCF7 cells.

### Binding to CAR-negative cells is integrin-dependent

**Figure 4:**
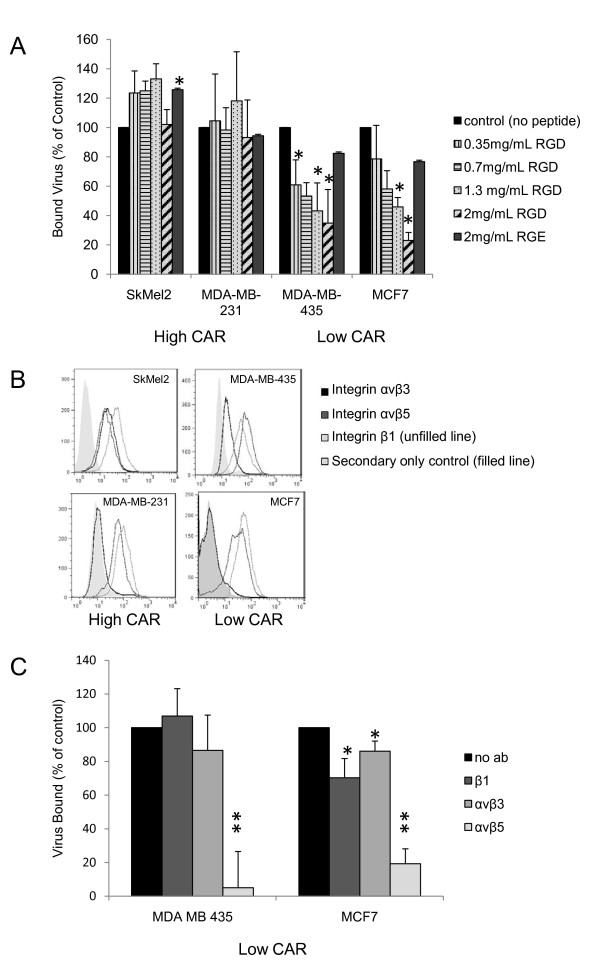
**The role of integrins in infection of low CAR cells**. (A) Cells were preincubated with media alone, increasing concentrations of synthetic peptide GRGDSP, or control peptide GRGESP for 1 hr followed by the addition of Ad5, all at 4°C for 6 hrs. Samples were then washed and fixed and virus bound was detected using an ELISA binding assay with an antibody to Ad5 as described in materials and methods. Data shown is the average of at least three independent experiments and error bars represent standard deviation. * indicates statistical significance, p ≤ 0.05. (B) Surface integrin levels were determined using flow cytometry. Cells were stained with LM609 (integrin αvβ3), P1F6 (integrin αvβ5), JB1A (integrin β1), or secondary only control, goat anti-mouse Alexa 488. The secondary only population was set to approximately 1% positive and used as the negative population for each cell line. The percentage of cells which are positive for each integrin as well as the MFI is quantified and displayed in Table 2. Data shown is representative of at least two independent experiments. (C) Cells were preincubated with media alone or the blocking antibodies LM609, P1F6, OR JB1A for 1 hr followed by the addition of Ad5, all at 4°C. Virus bound was determined as described in (A). Data shown is the average of at least three independent experiments and error bars represent standard deviation. * indicates statistical significance, p ≤ 0.05; ** indicates statistical significance, p ≤ 0.01.

Our fiber blocking studies ruled out the possibility that fiber binds an alternate receptor in CAR-negative cells. A second well-characterized interaction between Ad5 and the cell surface is the binding of the RGD (Arg-Gly-Asp) domain in the penton base of adenovirus to integrin αvβ3 and integrin αvβ5 [8]. Integrins are heterodimeric cell surface molecules that mediate cell-extracellular matrix and cell-cell interactions and are therefore involved in a number of cellular processes [38]. Additionally, several viruses and bacteria have been reported to use integrins to enter host cells [39-42]. Integrin-mediated processes are often regulated by both ligand binding and integrin clustering; therefore, many integrin ligands are multivalent, able to bind multiple integrins simultaneously [43]. The crystal structure of the RGD domain of the adenovirus penton base binding integrin αvβ5 has been resolved, revealing that one penton base complex of the virus binds approximately four integrin molecules [44]. Blocking binding to integrin αvβ5, as well as αvβ3, prevents adenovirus from being internalized but does not impact binding of adenovirus to the cell surface [8]. Although these studies predate the discovery of CAR, the cells used in them expressed a fiber receptor, most likely CAR, as infection of the cells could be blocked by soluble fiber [8]. Another study suggests integrin α3β1 can bind Ad5 to cells, both in the presence and absence of CAR, although the mechanism of this binding is unclear since it apparently is not mediated by the RGD domain of Ad5 penton base [45]. Additionally, Huang et al observed that in hematopoietic cells lacking CAR, binding to the cell surface is mediated by the leukocyte integrin αMβ2 [13]. Therefore, we sought to identify what role integrins play in the infection of these low CAR cancer cells. To distinguish between two possibilities, binding and internalization, we used an assay to directly measure binding. Cells were plated in 96-well plates and incubated overnight at 37°C. Cells were chilled to 4°C, a temperature which allows binding but does not permit internalization, and then preincubated with increasing concentrations of an integrin-blocking peptide, RGD, or a control peptide, RGE. Ad5 was added to the cells at 4°C and incubated for six hours. Cells were washed and fixed and virus bound determined using an antibody directed against Ad5 capsid proteins. Figure 4a shows that in SkMel2 and MDA-MB-231 cells, which express CAR, integrin blocking peptide RGD does not block Ad5 binding, consistent with previous reports. However, in both MDA-MB-435 and MCF7 cells, which are low CAR, Ad5 binding to cells is blocked by RGD, showing that binding in these cells is integrin-dependent.

αvβ3, αvβ5, and the β1 subunithave been specifically implicated in adenovirus infection [8,45-47]. Therefore, we measured the levels of these integrins on the surface of MDA-MB-435 and MCF7 cells, both of which lack CAR expression, as well as SkMel2 and MDA-MB-231 cells, both of which express CAR. We found that MDA-MB-435 and SkMel2 cells express all three integrins (Figure 4b). MCF7 and MDA-MB-231 cells only express αvβ5 and the β1 subunit (Figure 4b). The expression levels are quantified both by percentage of cells staining positive and by MFI in Table 2.

**Table 2:** Quantification of Integrins expression across panel of cell lines

	αVβ3 expression		αVβ5 expression		β1 expression	
**Cell Line**	**% positive**	**MFI**	**% positive**	**MFI**	**% positive**	**MFI**

SkMel2	79.5	28	69.9	26.6	99	52.8

MDA-MB-231	6.06	177	74.8	94.2	92.2	120

MDA-MB-435	6.28	107	94.8	110	99.4	68.3

MCF7	4.5	32.2	75.9	48.4	84.3	63.9

Quantification of data displayed in Figure 4b. MFI is mean fluorescence intensity.

Next, we investigated whether one of these integrins is responsible for the binding of Ad5 to low CAR cells. Again at 4°C to prevent virus internalization, cells were preincubated with antibodies that block ligand binding to integrins and then Ad5 was added to cells and the amount of virus bound measured. Preincubation with blocking antibodies to β1 or to αvβ3, in either MDA-MB-435 or MCF7 cells, did not inhibit infection by very much. However, blocking αvβ5 dramatically reduced Ad5 binding in both cell lines. In MDA-MB-435 cells, blocking αvβ5 reduced binding to only 5% of control and in MCF7 cells, binding is reduced to 19% of control (Figure 4c). From this result, we conclude that binding of Ad5 to low CAR cells occurs via the RGD domain of penton base binding to integrin αvβ5.

### Binding of Ad5 via integrin αvβ5 to CAR-negative cells is high affinity

**Figure 5:**
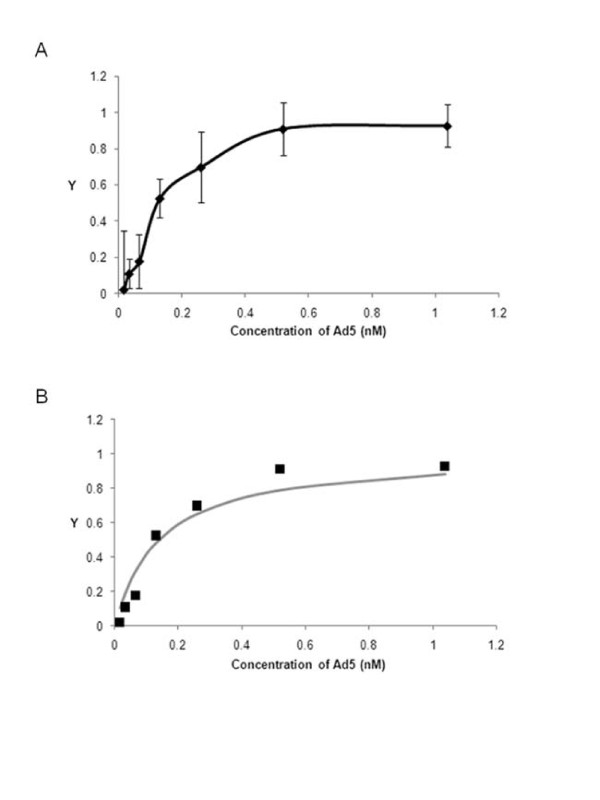
**Ad5 binding to cells via integrin αvβ5 is high affinity**. (A) Cells were incubated with various concentrations of Ad5 at 4°C for sufficient time for virus bound to reach equilibrium. Virus bound was measured as described in Figure 4. Y is the fractional occupancy, which is a ratio of virus bound to maximum virus bound. Data shown is the average of at least three independent experiments and error bars represent standard deviation. (B) Observed data (diamonds) was fit to calculated values (line) using the Langmuir Binding Isotherm (Eqn 1). K_d _was determined by Microsoft Excel's Solver function using non-linear regression analysis to solve Eqn 1. A K_d _of 1.4 × 10^-10 ^M was calculated from the observed values.

To further characterize the interaction between Ad5 and the surface of cells in which binding occurs via integrin αvβ5, we measured the binding affinity of whole Ad5 and the surface of MDA-MB-435 cells, which bind Ad5 through integrin αvβ5 (Figure 4c). Figure 5a shows binding to MDA-MB-435 cells is specific and saturable and represents a typical binding isotherm. To determine the dissociation constant (K_d_), a measure of the strength of an interaction, we fit the data to the Langmuir binding isotherm (Eqn 1) [48].(1)

Here, Y is the fractional occupancy of the receptor and [L] is the ligand concentration. We performed a non-linear least-squared analysis using MS Excel's Solver function to calculate the K_d_. Figure 5b shows observed versus calculated values of Y, demonstrating the observed values fit this equation. We calculate a K_D _of 1.4 × 10^-10 ^M. Therefore, we conclude that Ad5 can bind to cells via integrin αvβ5 and this interaction is high affinity, in the picomolar range. Since previous studies have demonstrated that one penton base can bind approximately 4 integrins, the multivalent nature of the penton base integrin interaction likely contributes to the high affinity observed [44].

## Discussion

In this study we report that cells which do not express CAR can be efficiently transduced by Ad5. Adenovirus entry is not dependent on fiber binding to cells but instead is blocked by an RGD peptide that interferes with the RGD domain on the adenovirus penton base binding cellular integrins. Further, we find that binding to low CAR cells is inhibited specifically by a blocking antibody to integrin αvβ5, demonstrating that integrin αvβ5 is required for Ad5 attachment to these cells. The binding mediated by integrin αvβ5 is extremely high affinity, in the picomolar range. Our data further challenges the prevailing model of adenovirus infection, in which binding to a primary receptor, CAR, is required in order for subsequent interactions between adenovirus and integrins to initiate viral entry.

Ad5 binding to low CAR cells is independent of fiber and instead depends on an interaction with integrin αvβ5. Therefore, Ad5 does not require an independent binding receptor to dock it to the cell before it can interact with internalization receptors. Other viruses are also reported to use both primary binding and internalization receptors. HIV-1 first binds to CD4 followed by binding to the chemokine receptors CCR5 or CXCR4, which trigger membrane fusion [49]. Binding to CD4 induces conformational changes in the HIV protein gp120, revealing the previously hidden binding site for its coreceptors [6]. Variants with mutations in gp120 allowing for direct interaction with coreceptors have been isolated *in vitro*; however, these variants are sensitive to neutralizing antibodies and therefore selected against *in vivo *[50]. Therefore, the role of CD4 binding in HIV-1 infection may be particularly critical in evading the immune system of the host.

Unlike HIV-1 binding to CD4, Ad5 binding to CAR does not induce conformational changes in viral proteins, thus facilitating subsequent entry steps [51]. Rather, CAR is thought to facilitate a high affinity interaction to fiber, thus docking the virus to the cell surface and allowing the subsequent interaction between the penton base and integrins to initiate internalization. Indeed, only the extracellular domain of CAR is required for CAR-mediated adenovirus entry [52]. However, previous studies have observed CAR-independent infection and the work in this paper demonstrates that Ad5 can bind directly to integrin αvβ5, previously identified as an internalization receptor [13,14,17,22,45]. Therefore, our results and the results of others suggest this initial binding step is not required for entry. Nevertheless, CAR binding is conserved in a number of adenovirus serotypes and the fiber-CAR interaction is one that is well characterized and is itself of high affinity [10,53]. Therefore, CAR binding clearly plays an important role in the adenovirus infection cycle. One possibility is that, even when CAR is not needed to enter cells, CAR functions as an exit receptor [54]. Walters et al report that when Ad5 lyses a cell, excess fiber is released and through binding to CAR, disrupts neighboring cell-cell junctions, allowing for release of the virus back to the apical surface where it may continue infecting cells [54]. Additionally, recent work has demonstrated that adenovirus binding to CAR may induce downstream signaling events that increase integrin activation, thus promoting infection[55]. Therefore, binding to CAR may facilitate entry in ways beyond simply docking the virus to the cell surface. Indeed, at least two serotypes of adenovirus, Ad9 and Ad37, have fibers which bind CAR but do not use CAR as an attachment receptor, supporting the idea that CAR binding is important for steps other than attachment [10,56]. Further, it is likely advantageous to the virus to be able to use multiple entry routes, enabled by its ability to engage multiple different receptors.

Both HIV, as evidenced by CD4-independent variants isolated *in vitro*, and Ad5, as evidenced by our results and the results of others can infect cells without binding to their so-called primary receptors. Binding to these receptors, instead of being strictly required for infection, may contribute to other necessary parts of the virus infection cycle, such as evading the host immune system or facilitating virus escape. Many other viruses with less characterized receptors seem to also use multiple receptors, some classified as binding receptors [57]. For example, rotaviruses are thought to first bind to a sialic acid (SA)-containing molecule, which anchors the virus to the cell, and then bind to coreceptors to initiate viral entry [57]. Mutant variants of rotaviruses that are SA-independent and interact directly with coreceptors have been isolated *in vitro*, suggesting that similarly to HIV and Ad5, binding to the primary receptor is not strictly required for infection [58,59]. Therefore, the interaction between rotaviruses and SA-containing molecules may facilitate an as yet unidentified aspect of rotavirus infection. As more functional roles of virus receptors in infection are elucidated, the use of binding receptors in other aspects of the viral life cycle may emerge as a general principle of viral pathogenesis.

In addition to being used as a model system for viral entry, much effort has been put into developing adenoviruses, especially species C adenoviruses including Ad5, as vectors for gene therapy. In fact, adenoviral vectors have been used in more than one quarter of gene therapy trials worldwide [60]. Cancer is one of the most common targets of adenovirus-mediated gene therapy. As mentioned previously, CAR expression is often lost as cancers progress and this loss has been viewed as a major hurdle to using adenovirus-based therapies in cancer [31,33-36]. However, integrin αvβ5 has been reported to be overexpressed in cancers [61,62]. Therefore, our conclusion that Ad5 can use integrin αvβ5 to bind to and infect cells lacking CAR suggests that cancer cells having lost CAR expression may still be good targets for adenovirus-based therapies. Recent work has shown that erythrocytes sequester adenovirus by binding CAR, thus limiting systemic infection; therefore, using CAR-ablated vectors, a strategy many groups are attempting, may improve delivery for gene therapy for reasons beyond changing receptor interactions [63,64]. We also observed what may be an as yet unidentified obstacle to these therapies, however. T47D cells, which express CAR (Figure 2a) and integrin αvβ5 (data not shown) are still resistant to Ad5 infection (Figure 1). Wang et al showed the cellular protein CEACAM6 blocks adenovirus trafficking to the nucleus in human pancreatic cancer cell lines[65]. Future studies to determine if this protein blocks infection in T47D cells, or if resistance is due to a novel mechanism are needed.

## Conclusions

In conclusion, we have found that cells which express little to no CAR can still be efficiently transduced by Ad5. In these cells, CAR is not required: Binding occurs through integrin αvβ5. We show for the first time that integrin αvβ5, previously described as an internalization receptor, can also be used as an alternate binding receptor for Ad5.

## Methods

### Cell Lines and Viruses

SkMel2 and WM278 cells are human melanoma cell lines. MCF7, MDA-MB-453, BT549, T47D, MDA-MB-435, and MDA-MB-231 are human breast cancer cell lines. All cells were obtained from the ATCC. SkMel2 cells were cultured in MEM supplemented with sodium pyruvate, non-essential amino acids, and 10% FBS. WM278 were cultured in DME-H16 and supplemented with 10% FBS. All other cancer cell lines were cultured in DMEM supplemented with 10% FBS. Virus used was replication incompetent E1A deleted and expressed GFP (Ad5-GFP). Virus was propagated in HEK293/E4/pIX cells and harvested by CsCl gradient ultracentrifugation as previously described [66,67]. Virus titers were determined as previously described [68].

### Antibodies and Peptides

The MAb RmcB was used to detect CAR expression [12]. The MAbs LM609, P1F6, and JB1A directed against integrins αvβ3, αvβ5, and the β1 subunit respectively were purchased from Chemicon. The secondary antibody Alexa 488 was purchased from Molecular Probes. The synthetic peptides GRGDSP and GRGESP were purchased from Sigma. All antibodies were IgG.

### Recombinant Fiber

Full length Ad5 fiber was cloned from a Gateway entry vector into a his-tagged destination vector using the Gateway system per manufacturer's instructions (Invitrogen). Fiber was transformed into and grown up in BL21 Star (DE3) *E.coli *(Invitrogen). Overnight starter culture was diluted 1:100 in LB/amp and grown until bacteria reached log phase. 50 uM IPTG was added and bacteria were grown at room temperature overnight. Pellets were disrupted using Bugbuster (Novagen) per manufacturer's instructions. Fiber was purified via its his-tag by incubation with Probond resin (Invitrogen), several washes with 20 mM Imidizole, and elution using Poly-Prep Chromatography Columns (BioRad) with 0.2 M Imidizole. Purified recombinant fiber was then dialyzed into PBS for use in experiments. Approximately 1 mg/L of purified fiber was obtained.

### Cell Infection Assay

8 × 10^5 ^cells were plated in 6-well plates and incubated overnight at 37°C. Cells were infected with Ad5-GFP at MOI 25 in DMEM with 2% FBS. After overnight incubation at 37°C, cells were trypsinized, washed with PBS, and GFP expression quantitated using flow cytometry. The BD FACSCalibur Flow Cytometer is the instrument used, 10,000 events were acquired for each experiment, gating for live cells, and FlowJo software was used to generate histograms and analyze data. For fiber blocking experiment, prior to addition of Ad5-GFP, different quantities of soluble fiber (1 ug/mL, 5 ug/mL, or 25 ug/mL) were added to cells, incubated at room temperature for 1 hr. Then Ad5-GFP was added to cells at MOI 25 and cells were incubated overnight at 37°C before flow cytometry analysis.

### Surface expression levels

Cells were trypsinized, washed with PBS, and 1 × 10^6 ^cells were incubated with primary antibody for 30 minutes on ice. Cells were washed, incubated with secondary for 30 minutes on ice, and analyzed by flow cytometry. The BD FACSCalibur Flow Cytometer is the instrument used, 10,000 events were acquired for each experiment, gating for live cells, and FlowJo software was used to generate histograms and analyze data. Dilutions were as follows: RmcB (1-50), LM609 (1-100), P1F6 (1-100), JB1A (1-100), Alexa 488 (1-100). Secondary antibody (Alexa 488) alone was used as a control for each cell line.

### Statistics

Microsoft Excel was used to do a 2 tailed, type 3 T Test to determine statistical significance.

### Quantitative PCR

Total RNA was isolated from cells using RNeasy Mini Kit (Qiagen). PCR was performed by the Genome Analysis Core Facility, Helen Diller Family Comprehensive Cancer Center, University of California, San Francisco. PCR was conducted in triplicate with 20 uL reaction volumes of 1× TaqMan buffer (1× Applied Biosystems PCR buffer, 20% glycerol, 2.5% gelatin, 60 nM Rox as a passive reference), 5.5 mM MgCl_2_, 0.5 mM each primer, 0.2 uM each deoxynucleotide triphosphate (dNTP), 200 nM probe, and 0.025 unit/uL AmpliTaq Gold (Applied Biosystems) with 5 ng cDNA. A large master mix of the above-mentioned components (minus the primers, probe, and cDNA) was made for each experiment and aliquoted into individual tubes, one for each cDNA sample. cDNA was then added to the aliquoted master mix. The master mix with cDNA was aliquoted into a 384-well plate. The primers and probes were mixed together and added to the master mix and cDNA in the 384-well plate. PCR was conducted on the ABI 7900HT (Applied Biosystems) using the following cycle parameters: 1 cycle of 95° for 10 minutes and 40 cycles of 95° for 15 seconds, 60° for 1 minute. Analysis was carried out using the SDS software (version 2.3) supplied with the ABI 7900HT to determine the Ct values of each reaction. Ct values were determined for three test and three reference reactions in each sample, averaged, and subtracted to obtain the ΔCt [ΔCt = Ct (test locus) – Ct (control locus)]. PCR efficiencies were measured for all custom assays and were greater than or equal to 90%. Therefore, relative fold difference was calculated for each primer/probe combination as 2^-ΔCt ^× 100. PCR primer and TaqMan probe sequences were synthesized by Integrated DNA Technologies (Coralville, IA) [or purchased from Applied Biosystems]. The sequences were as follows

Human CAR

Amplicon:

GGCGCTCCTGCTGTGCTTCGTGCTCCTGTGCGGAGTAGTGGATTTCGCCAGAAGTTTGAGTATCACTACTCCTGAAGAGATGATTGAAAAAGCCAAAG

Forward: GGCGCTCCTGCTGTGC

Reverse: CTTTGGCTTTTTCAATCATCTCTTC

Probe: TGCGGAGTAGTGGATTTCGCCAGAAG

Human GapDH:

Amplicon: ATTCCACCCATGGCAAATTCCATGGCACCGTCAAGGCTGAGAACGGGAAGCTTGTCATCAATGGAAATCCCA

Forward: ATTCCACCCATGGCAAATTC

Reverse: TGGGATTTCCATTGATGACAAG

Probe: ATGGCACCGTCAAGGCTGAGAACG

### Ad5 Binding Assay

Cells were plated in 96-well SigmaScreen poly-D-lysine coated plates (Sigma) and incubated overnight at 37°C. For peptide and antibody blocking experiments, cells were prechilled at 4°C for 30 minutes followed by addition of either peptide at indicated concentration or antibody (500 ug/mL) for 1 hr. Ad5-GFP (0.04 ug/uL, protein concentration determined by Bradford assay) diluted in DMEM with 50% FBS was added to cells and incubated for 6 hrs at 4°C. Cells were washed several times and fixed with ice cold solution of 95% EtOH/5% Acetic Acid. Cells were washed 1× TBST (0.05M Tris, 0.15 M NaCl, 0.5% Tween-20, pH 7.5) and incubated in Superblock (Pierce) for 1 hr at RT. Cells were washed 2× Superblock followed by incubation with a non-related control IgG antibody to block any non-specific interactions for 30 min, RT. Cells were washed 1× TBST and incubated with polyclonal rabbit anti-Ad5 antibody (Access Biomedical) for 30 min, RT, followed by washing 4xTBST. Cells were next incubated with Goat-anti-Rabbit-AP (Pierce) for 30 min, RT, followed by washing 4xTBST. Signal was then amplified and detected using an Elisa Amplification System per manufacturer's instructions (Invitrogen). For determining K_D _of Ad5 binding to cells, the above protocol was used except cells were incubated with Ad5 at varying concentrations for 18 hrs at 4°C prior to fixing

## Competing interests

The authors declare that they have no competing interests.

## Authors' contributions

CL carried out the experiments, participated in the design and coordination of the study, and drafted the manuscript. FM participated in the design and coordination of the study and contributed to the writing of the manuscript. All authors read and approved the final manuscript.

## Supplementary Material

Additional file 1 **Dose response of Ad5 infection in a high CAR cell line and a low-CAR cell line**. Cells were infected with Ad5-GFP at the indicated MOI and incubated overnight. Infection was quantified using flow cytometry analysis to quantify infection at each MOI in each cell line. The mean fluorescence intensity is quantified and displayed in the figure. Data shown is representative of at least two independent experiments. Click here for file
