# Supplementary figures and images for: Integrin αvβ5 is a primary receptor for adenovirus in CAR-negative cells
Source: Virol J. 2010 Jul 8;7:148. doi: 10.1186/1743-422X-7-148 (PMC2909962; doi:10.1186/1743-422X-7-148)

A

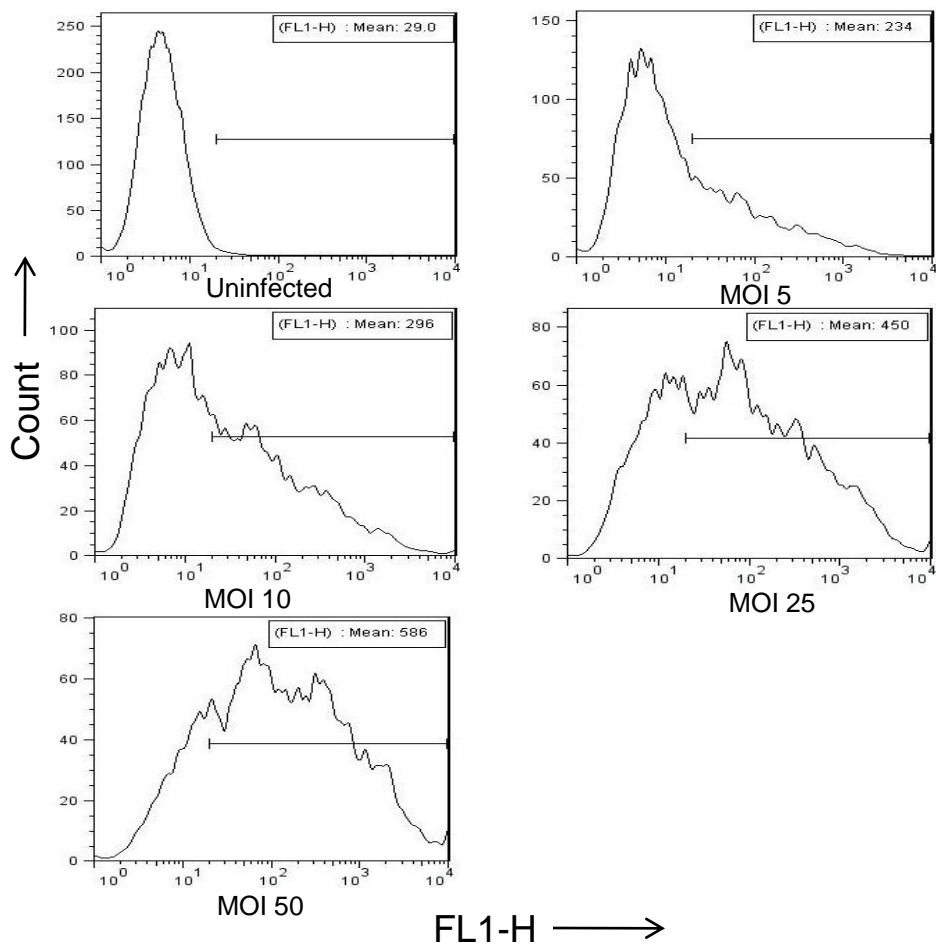

B

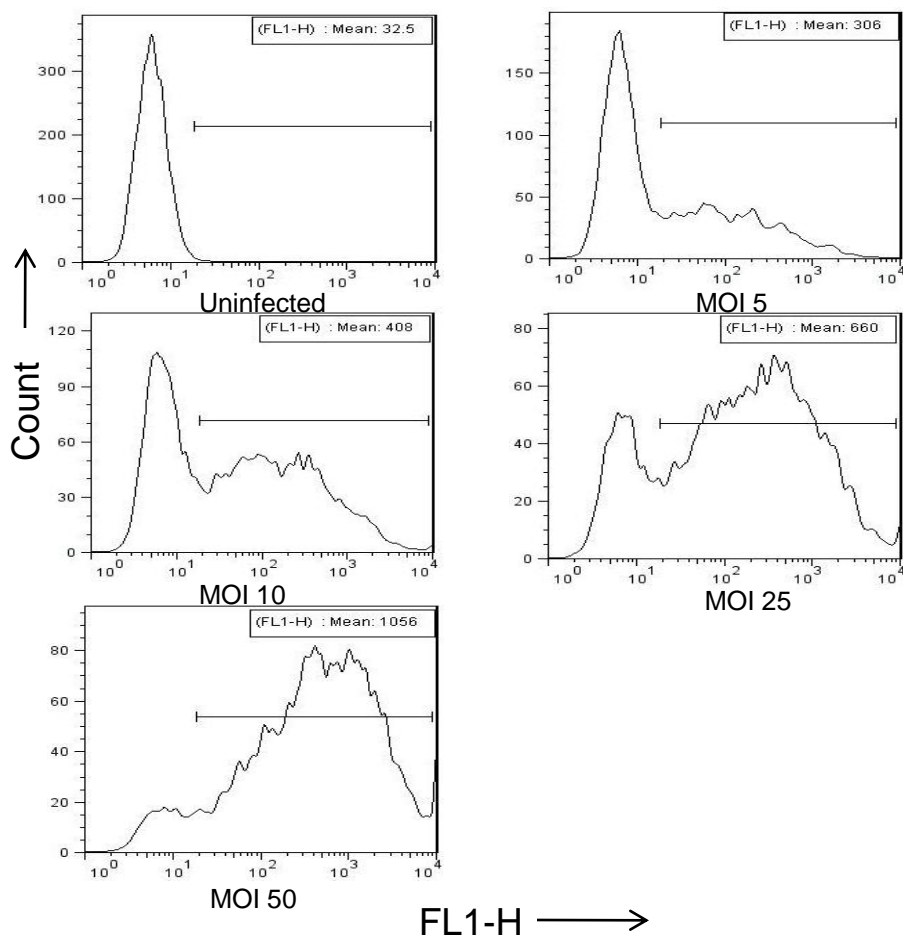

Supplement: Additional file 1 — Dose response of Ad5 infection in a high CAR cell line and a low-CAR cell line. Cells were infected with Ad5-GFP at the indicated MOI and incubated overnight. Infection was quantified using flow cytometry analysis to quantify infection at each MOI in each cell line. The mean fluorescence intensity is quantified and displayed in the figure. Data shown is representative of at least two independent experiments. [file 1743-422X-7-148-S1.PDF]
